# Association between psoas muscle area and outcomes after transcatheter tricuspid valve repair

**DOI:** 10.1007/s12928-025-01136-3

**Published:** 2025-05-27

**Authors:** Mahmoud Balata, Tetsu Tanaka, Atsushi Sugiura, Refik Kavsur, Johanna Vogelhuber, Can Öztürk, Sebastian Zimmer, Julian Luetkens, Georg Nickenig, Marcel Weber

**Affiliations:** 1 Heart Center Bonn, Department of Internal Medicine II, University Hospital Bonn, Venusberg-Campus 1, 53127 Bonn, Germany; 2 Department of Cardiology, St. Marianna University School of Medicine, Kawasaki, Japan; 3 Department of Radiology, University Hospital Bonn, Bonn, Germany

**Keywords:** Transcatheter tricuspid valve repair, Tricuspid regurgitation, Psoas muscle area, Sarcopenia

## Abstract

Transcatheter tricuspid valve repair (TTVR) might be an alternative option to reduce tricuspid regurgitation (TR) in patients with sarcopenia. Psoas muscle area (PMA) measured using computed tomography (CT) is known as a marker of sarcopenia. We investigated the association of PMA with procedural and clinical outcomes following TTVR. We retrospectively measured left and right PMA using pre-procedural CT in patients undergoing TTVR. Low PMA was defined as the total PMA < 20.3 cm^2^ for men and < 11.8 cm^2^ for women. The primary outcome was a composite of all-cause mortality and heart failure hospitalization within one year after TTVR. Of 163 patients, 47 (29%) were considered as having low PMA. Procedural success, defined as residual TR of ≤ 2 + at discharge, and in-hospital mortality were comparable between patients with low and high PMA. Patients with low PMA had a higher incidence of the composite outcome than those with high PMA (49% vs. 21%; p = 0.001) This association was consistent after adjusting for baseline characteristics (adjusted hazard ratio 0.43; 95% confidence interval: 0.23–0.80; p = 0.008). In contrast, the New York Heart Association functional class improved from baseline to three-month follow-up, regardless of PMA. Approximately 30% of patients undergoing TTVR were found to have low PMA. Although TTVR may be a safe therapeutic option to reduce TR and improve heart failure symptoms in patients with sarcopenia, the prognosis after TTVR remains poor in this population. Pre-procedural CT-based assessment of PMA may enhance risk stratification and support better clinical decision-making for TTVR.

**Graphical abstract:**

Psoas muscle area and outcomes after transcatheter tricuspid valve repair Patients with low psoas muscle area (PMA) had a higher incidence of all-cause mortality (upper right) and heart failure hospitalization (lower right). CT = computed tomography.

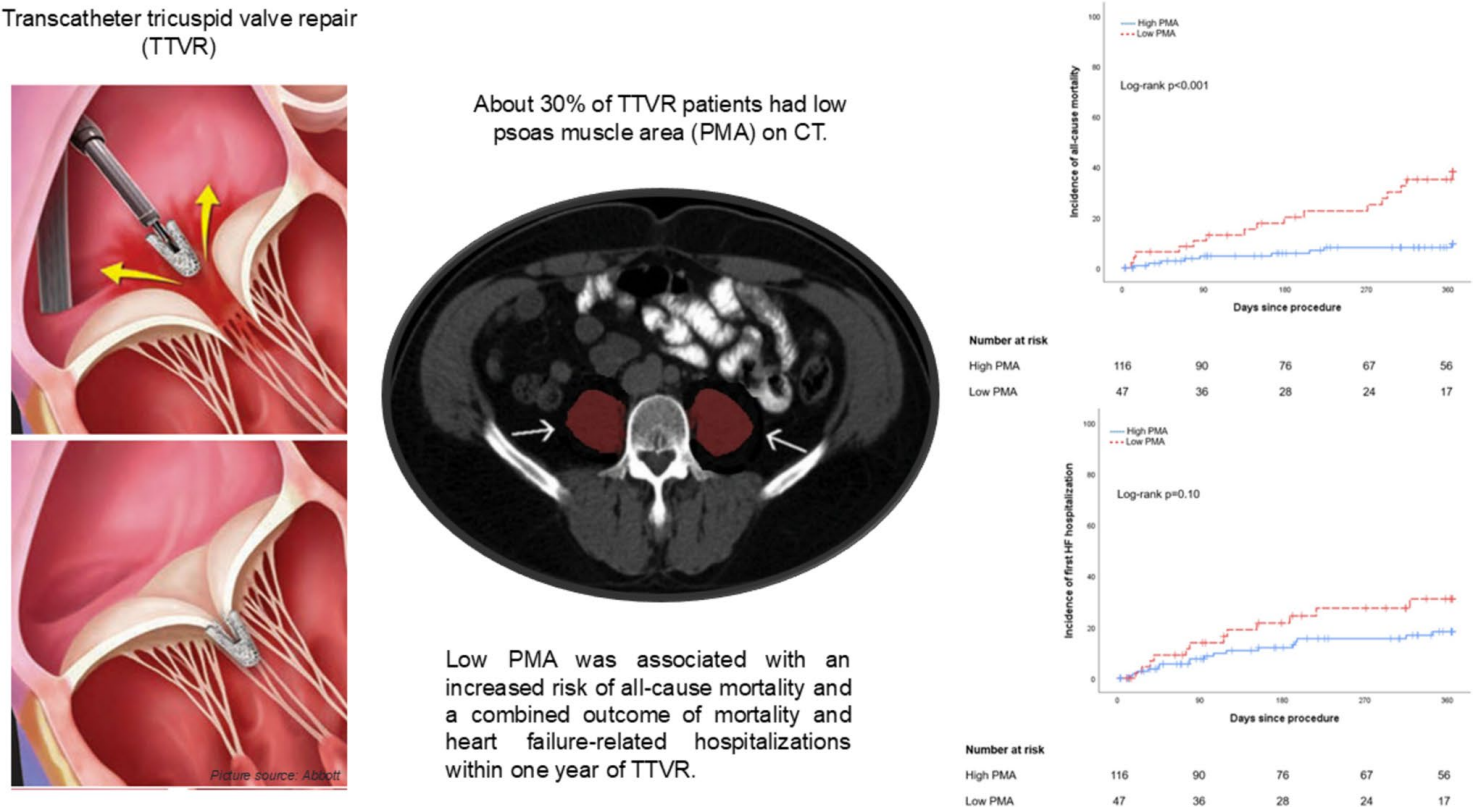

**Supplementary Information:**

The online version contains supplementary material available at 10.1007/s12928-025-01136-3.

## Introduction

Tricuspid regurgitation (TR) is associated with worsening heart failure (HF) symptoms and an elevated risk of mortality and morbidity [1, 2]. Although tricuspid valve surgery has been the first-line therapy for patients with TR, the isolated tricuspid valve surgery shows a high peri-procedural mortality risk and may not improve clinical outcomes of patients with TR [3–6]. On the other hand, transcatheter techniques may be a promising alternative for patients with TR. Transcatheter tricuspid valve repair (TTVR) may be effective in reducing TR and improving quality of life, with exhibiting a low peri-procedural mortality rate [7, 8].

Sarcopenia is an age-related, progressive, systemic musculoskeletal disorder characterized by loss of muscle mass and strength and is related to frailty and diminished functional capacity [9]. Sarcopenia is estimated to be concomitant in nearly half of heart failure patients, with aggravating clinical prognosis of the patients and elevating the risk of peri-procedural adverse events after cardiovascular interventions [10]. In the setting of tricuspid regurgitation, approximately one-third of patients undergoing isolated tricuspid valve surgery have sarcopenia, which may contribute to an increased risk of peri-procedural adverse events after tricuspid valve surgery [11]. Since the safety of TTVR has been proven in the recent clinical trials, the less-invasive transcatheter treatments might be a good alternative option for TR patients with sarcopenia. Understanding the procedural and clinical outcomes of TTVR in patients with sarcopenia is an essential step toward improving the prognosis of this population.

Imaging assessment of muscle mass using computed tomography (CT) may assist with the detection of sarcopenia [9]. In particular, psoas muscle area (PMA) measured by CT is a simple parameter of the loss of muscle mass, with predictive value for clinical outcomes [9, 12–14]. The assessment of PMA using CT images might help the detection of sarcopenia in patients undergoing TTVR and the risk stratification for the intervention. Therefore, we measured PMA using peri-procedural CT scans in patients undergoing TTVR and investigated the association of PMA with clinical outcomes after TTVR.

## Methods

### Study population

This study was designed as a retrospective analysis using data from the Bonn registry, which is a prospective, consecutive collection of patients at the Heart Center Bonn, University Hospital Bonn, Germany. We identified symptomatic patients who underwent TTVR between August 2015 and May 2022. A standardized diagnostic workup was performed on all patients, including transesophageal echocardiography, and left- and right-heart catheterization. The decision to perform the intervention was made by the interdisciplinary heart team. For this study, we included only patients who underwent pre-procedural CT prior TTVR. Ethical approval for the Bonn Registry was obtained from the Local Ethics Committee of the University of Bonn. The study was conducted in accordance with the Declaration of Helsinki and its subsequent amendments, and all patients provided written informed consent.

### CT assessment of psoas muscle area

Pre-procedural CT images were analyzed by duplicate observers using the CoreSlicer web-based software, as previously reported [15]. Left and right PMAs were semi-automatically measured using an axial CT image at the top of the L4 vertebra. Although optimal cutoff values for low PMA using CT imaging have not yet been established, we defined low PMA as a total PMA of < 20.3 cm^2^ for men and < 11.8 cm^2^ for women in this study. This definition is based on a previous study by Mamane et al., in which the prevalence and prognostic impact of low PMA was evaluated in older patients undergoing transcatheter valve interventions [16].

### Echocardiographic assessments

Echocardiography was performed at baseline and before discharge based on the current guidelines [17]. The severity of TR was evaluated using a 5-grade scale based on qualitative and semi-quantitative measurements [18].

### Procedure

The TTVR procedures were performed using the MitraClip or TriClip system (Abbott Structural Heart, Santa Clara, California, USA), PASCAL system (Edwards Lifesciences, Irvine, California, USA), or Cardioband system (Edwards Lifesciences). Technical success was defined as a successful implantation of the intended devices in the tricuspid valve position with retrieval of the delivery system. Procedural success was defined as the successful implantation of devices with a reduction of TR severity to ≤ 2 + upon discharge. Periprocedural bleeding was identified as any bleeding that occurred within 48 h of the procedure. Acute kidney injury (AKI) was defined as an absolute increase in serum creatinine of ≥ 0.3 mg/dl or a relative increase of ≥ 50% from baseline to 48 h after the procedure [19].

### Outcome measures

The primary outcome was a composite of all-cause mortality and hospitalization due to heart failure within one year after TTVR. Secondary endpoints were in-hospital mortality and each single component of the composite outcome. New York Heart Association (NYHA) functional class was collected three months after TTVR. All clinical events, including hospitalization due to heart failure, were independently adjudicated by the local heart team based on the criteria of the Valve Academic Research Consortium [20]. The data of clinical events was collected from admission and outpatient medical records, interviews on the telephone, or documentation from the referring general practitioners.

### Statistical analysis

Categorical variables are presented as counts with percentages, and continuous variables are presented as either the mean ± standard deviation or the median with an interquartile range (IQR). Continuous variables were compared between two groups using the t-test or the Mann–Whitney U test. Categorical variables were compared between the groups using the chi-square or Fischer exact tests. Inter- and inrtra-observer agreements were assessed in randomly selected 20 cases using intraclass correlation coefficients (ICC). NYHA class was compared between baseline and the follow-up using Wilcoxon signed-rank test.

Time-to-event curves were generated using the Kaplan–Meier method and were compared between groups using the log-rank test. Hazard ratios (HRs) and corresponding 95% confidence intervals (CIs) for the outcomes were calculated using univariate and multivariable Cox proportional hazard models. The multivariable model included predetermined covariates based on their presumed associations with clinical outcomes: age, sex, estimated glomerular filtration rate, chronic obstructive pulmonary disease, left ventricular ejection fraction (LVEF), and tricuspid annular plane systolic excursion (TAPSE). As a sensitivity analysis, we conducted another multivariable model including TRI-SCORE [21]. The incremental value of adding low PMA to TRI-SCORE was evaluated using the receiver-operating characteristic analysis. Statistical significance was defined as a two-sided p-value < 0.05. The statistical analysis was conducted using IBM SPSS Statistics version 26.0 software (IBM Corp, Armonk, NY, USA).

## Results

### Study population

Between August 2015 and May 2022, a total of 339 patients underwent TTVR. Of these, 163 patients with pre-procedural CT images covering the abdominal region were included in the present analysis (Supplemental Figure S1). Comparison of baseline characteristics between patients with and without pre-procedural CT images is shown in Supplemental Table S1.

Overall, the mean age was 79 ± 7 years, 45% of the study participants were male, and the median BMI was 24.6 kg/m^2^ (IQR: 22.0–27.2 kg/m^2^). Most of the study participants were considered as having a high surgical risk (TRI-SCORE: 5 points [IQR: 3–6 points]; EuroSCORE II: 5.5% [IQR: 3.2–11.5%]). The baseline echocardiography showed that 83 patients (51%) had massive or torrential TR. The median LVEF was 57% (IQR: 52–62%), and the mean TAPSE was 19 ± 5 mm.

### CT assessment of PMA

**Fig. 1:**
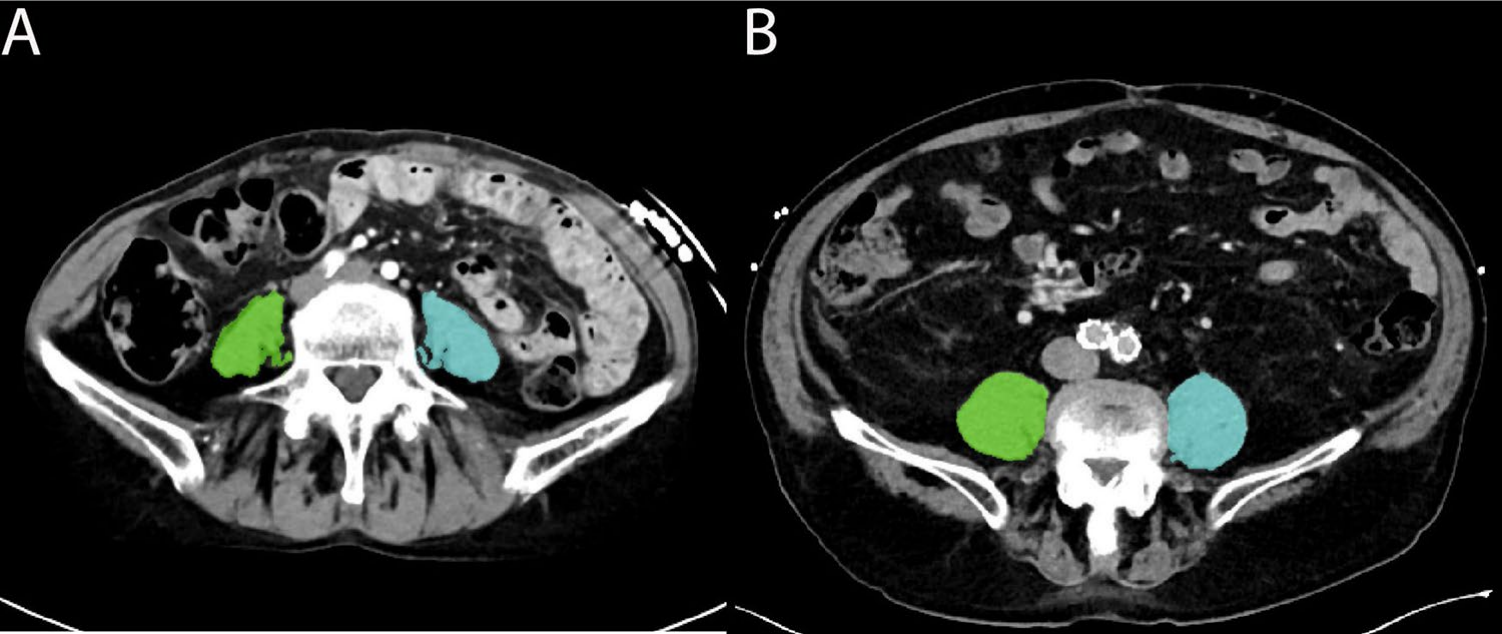
Assessment of psoas muscle area using computed tomography. Axial computed tomography (CT) images showing patients with low psoas muscle area (**A**) and high psoas muscle area (**B**)

The pre-procedural CT scans were performed at a median of 48 days (IQR: 35–95 days) prior to the TTVR procedure. The median value of total PMA was 17.9 cm^2^ (IQR: 14.1–21.4 cm^2^). Of the 163 patients, 47 (29%) were considered as having low PMA (Fig. 1**)**. The inter- and intra-observer agreements were excellent with intraclass correlation coefficient of 0.998 (p < 0.001) and 0.992 (p < 0.001) respectively.

Patients with low PMA were more likely to be male and to have renal dysfunction requiring hemodialysis, compared to those with high PMA (Table 1). In contrast, TR severity, TAPSE, and LVEF were comparable between the groups.

**Table 1:** Baseline characteristics

	Total	High PMA	Low PMA	p-value
	n = 163	n = 116	n = 47	
Age, year	80 (76–84)	80 (76–83)	81 (75–85)	0.58
Male	73 (45)	38 (33)	35 (75)	< 0.001
BMI, kg/m^2^	24.6 (22.0–27.2)	24.8 (22.1–28.1)	23.0 (20.9–26.1)	0.27
EuroSCORE II, %	5.5 (3.2–11.5)	4.8 (2.9–9.3)	9.7 (4.1–14.4)	0.004
TRI-SCORE, point	5 (3–6)	5 (3–6)	5 (4–6)	0.40
Diabetes mellitus	33 (20)	24 (21)	9 (20)	1.00
Arterial hypertension	135 (83)	98 (85)	37 (79)	0.37
Coronary artery disease	80 (49)	58 (50)	22 (47)	0.73
Previous myocardial infarction	28 (17)	21 (18)	7 (15)	0.62
Previous cardiac surgery	69 (42)	45 (39)	24 (51)	0.17
Previous stroke	12 (7)	9 (8)	3 (6)	1.00
Atrial fibrillation	150 (92)	105 (91)	45 (96)	0.35
NYHA functional class				0.13
II	37 (23)	28 (24)	9 (19)	
III	109 (67)	79 (69)	30 (64)	
IV	16 (10)	8 (7)	8 (17)	
Cardiac implantable electrical device	38 (23)	25 (22)	13 (28)	0.42
Hemoglobin, mg/dl	11.4 ± 1.9	11.4 ± 1.9	11.1 ± 1.9	0.38
eGFR, ml/min/m^2^	51 (35–64)	50 (35–66)	53 (37–63)	0.73
Hemodialysis	5 (3)	1 (1)	4 (9)	0.03
NT-proBNP, pg/ml	2225 (1283–4094)	2114 (1157–3765)	2501 (1584–5907)	0.19
Furosemide equivalent dose, mg/day	40 (20–80)	40 (20–75)	40 (20–80)	0.58
Echocardiographic assessment				
LVEF, %	57 (52–62)	58 (54–63)	56 (51–60)	0.12
LV end-diastolic volume index, ml/m^2^	39 (28–51)	35 (28–49)	44 (30–63)	0.23
LV end-systolic volume index, ml/m^2^	15 (11–24)	15 (10–22)	20 (13–27)	0.11
RA area, cm^2^	30 (24–35)	30 (24–35)	30 (25–37)	0.11
Tricuspid annulus diameter, mm	44 ± 7	43 ± 7	45 ± 8	0.20
RV mid-ventricular diameter, mm	38 (31–43)	37 (31–43)	39 (32–42)	0.25
Secondary TR	149 (91)	109 (94)	40 (85)	0.12
Severity of TR				0.66
Severe	80 (49)	58 (50)	22 (47)	
Massive	68 (42)	48 (41)	20 (43)	
Torrential	15 (9)	10 (9)	5 (11)	
Vena contracta, mm	10 (8–13)	10 (8–12)	10 (8–14)	0.91
EROA, mm^2^	64 (45–98)	60 (45–89)	77 (51–102)	0.16
TAPSE, mm	19 ± 4.9	19 ± 4.7	19 ± 5.3	0.93
RVFAC, %	45 ± 10	45 ± 9	43 ± 11	0.12
SPAP, mmHg	46 ± 15	46 ± 13	48 ± 17	0.26
CT parameter				
Left psoas area, cm^2^	9.1 (7.4–10.8)	9.5 (7.8–11.6)	8.5 (5.8–9.5)	< 0.001
Right psoas area, cm^2^	9.0 (7.3–10.7)	9.3 (7.3–11.2)	8.5 (6.5–9.7)	0.005
Total psoas area, cm^2^	17.9 (14.1–21.4)	18.8 (14.6–22.8)	16.8 (11.4–19.1)	< 0.001

Values are either the number (%), mean ± SD, or median (interquartile range)

*PMA* psoas mass area, *COPD* chronic obstructive pulmonary disease, *BMI*, body mass index, *NYHA* New York Heart Association, *eGFR* estimated glomerular filtration rate, *NT-proBNP* N-terminal pro-B-type natriuretic peptide, *LVEF* left ventricular ejection fraction, *RA* right atrium, *RV* right ventricle, *TR* tricuspid regurgitation, *EROA* effective regurgitant orifice area, *TAPSE* tricuspid annular plane systolic excursion; *RVFAC* right-ventricular fractional area change, *SPAP* systolic pulmonary artery pressure

### Procedural outcomes of TTVR

**Fig. 2:**
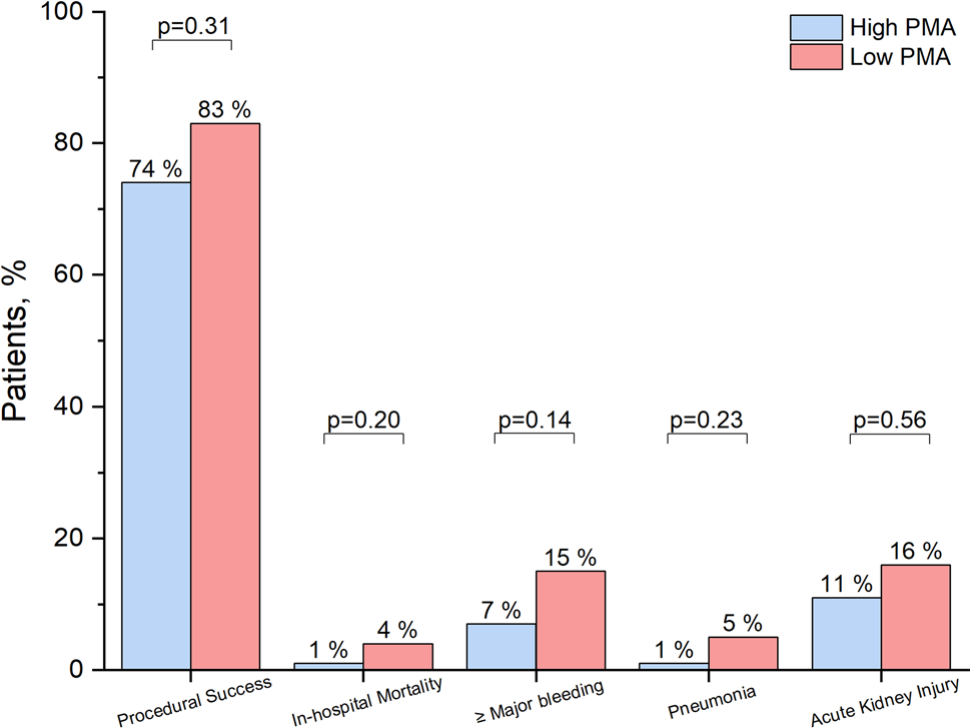
Procedural results and in-hospital adverse events

Most of the TTVR procedures were performed using transcatheter edge-to-edge repair devices (96%) (Table 2). No surgical conversion was required. Procedural success was achieved in 83% of patients with low PMA, which was comparable with patients with high PMA (74%; p = 0.31). There was no significant difference in in-hospital mortality between patients with low and high PMA (4% vs. 1%; p = 0.20), nor in the incidence of bleeding and acute kidney injury (Fig. 2). Furthermore, the length of post-procedural stay at hospital was comparable between patients with low and high PMA (5 days [IQR: 3–7 days] vs. 5 days [IQR: 4–7 days]; p = 0.34).

**Table 2:** Procedural and post-procedural findings

	Total	High PMA	Low PMA	p-value
	n = 163	n = 116	n = 47	
Device type				0.11
Edge-to-edge repair	157 (96)	110 (95)	47 (100)	
Annuloplasty	6 (4)	6 (5)	0 (0)	
Number of implanted clips	2 (1–2)	2 (1–2)	2 (1–2)	0.71
Technical success	150 (93)	107 (94)	43 (92)	0.73
Postprocedural hospitalization duration, days	5 (4–7)	5 (4–7)	5 (3–7)	0.34
Echocardiography at discharge				
Severity of TR				0.48
0	9(6)	5 (4)	4 (9)	
1+	56 (34)	41 (35)	15 (32)	
2+	60 (37)	40 (35)	20 (43)	
3+	29 (18)	24 (21)	5 (11)	
4+	9 (6)	6 (5)	3 (6)	
5+	0 (0)	0 (0)	0 (0)	
Procedural success	125 (77)	86 (74)	39 (83)	0.31
Tricuspid pressure gradient, mmHg	2.3 (1.6–2.8)	2.3 (1.6–2.8)	2.2 (1.7–2.9)	0.83

Values are either the number (%), mean ± SD, or median (interquartile range)

Abbreviations are shown in Table 1

### Association of PMA with clinical outcomes

The median follow-up length was 322 days (IQR: 94–365 days). Within one year, 25 patients (15%) died, and 29 (18%) were readmitted due to heart failure. Consequently, a total of 47 patients (29%) experienced a composite outcome within one year.

**Fig. 3:**
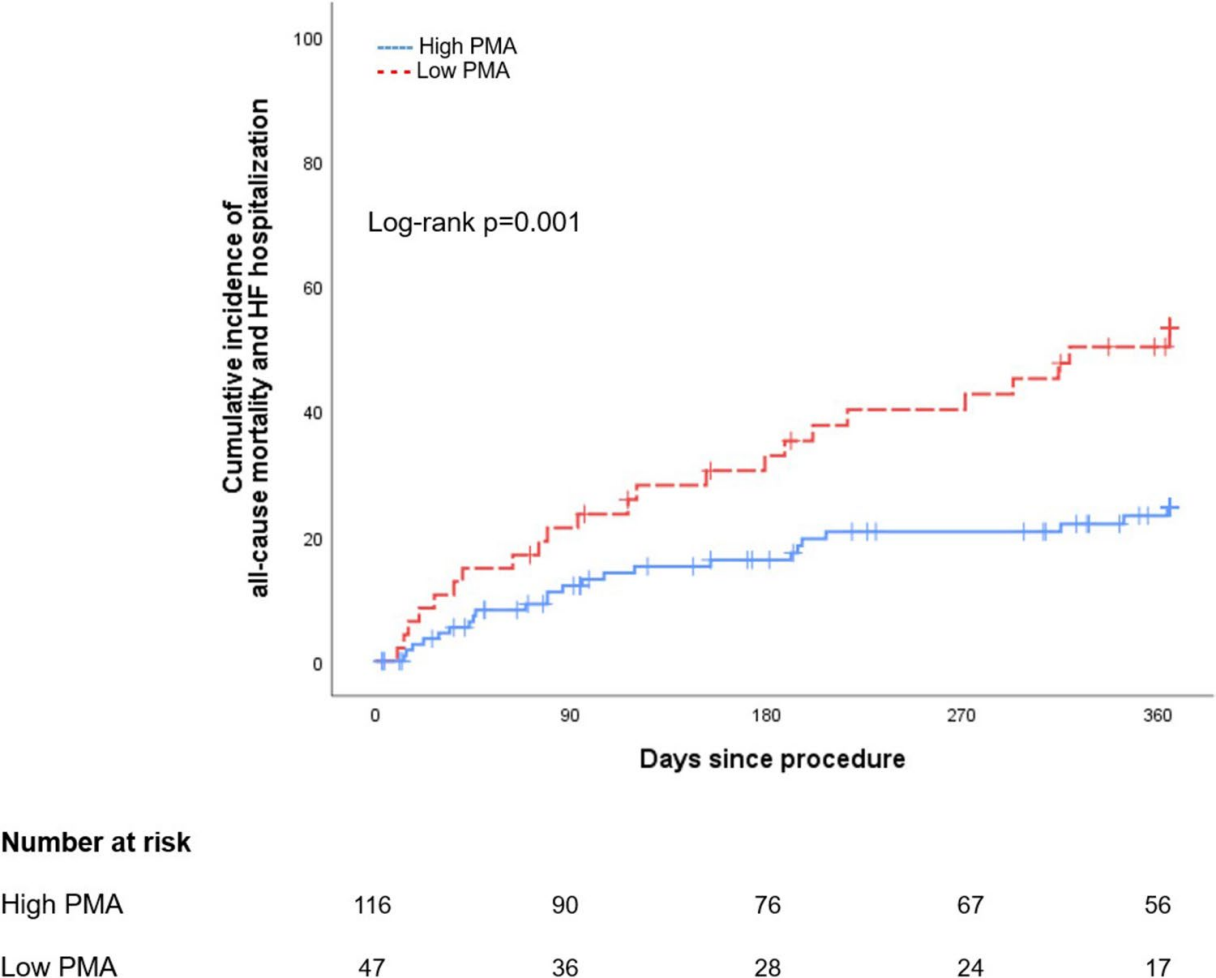
Incidence of the composite outcome according to psoas muscle area

Patients with low PMA had a higher incidence of the composite outcome than those with high PMA (49% vs. 21%; p = 0.001; Fig. 3). In the Cox proportional hazard analysis, low PMA was associated with a higher risk of the composite outcome within one year, irrespective of baseline characteristics (adjusted HR: 7.73; 95% CI 1.02–58.46; p = 0.048; Table 3 and Supplemental Table S2). Also, this association was consistent after adjusting for TRI-SCORE (adjusted HR: 2.35; 95% CI 1.32–4.17; p = 0.004; Supplemental Table S3).

**Table 3:** Association of PMA with the composite outcome within one year

	Univariate analysis			Multivariable analysis		
	HR	95%CI	p-value	HR	95%CI	p-value
Low PMA	2.52	1.42–4.47	0.002	2.15	1.13–4.10	0.02
Age, year	0.98	0.94–1.02	0.31	1.02	0.97–0.07	0.50
Male sex	1.70	0.95–3.02	0.07	1.36	0.70–0.63	0.37
eGFR, ml/min/m^2^	0.98	0.97–1.00	0.008	0.98	0.97–1.00	0.04
COPD	2.08	1.10–3.95	0.03	1.77	0.83–3.77	0.14
LVEF, %	0.97	0.95–0.99	0.02	0.97	0.94–1.00	0.08
TAPSE, mm	0.92	0.86–0.98	0.007	0.92	0.86–0.98	0.01

Abbreviations are shown in Table 1

The incidences of each component of the composite outcome are provided in Supplemental Figure S2. Patients with low PMA had a higher incidence of all-cause mortality than those with high PMA (34% vs. 8%; p < 0.001). Similarly, the incidence of hospitalization due to heart failure tended to be higher in patients with low PMA compared to those with high PMA (26% vs. 15%; p = 0.12).

Incremental value of PMA beyond TRI-SCORE to predict clinical outcomes after TTVR:

TRI-SCORE was related to the risk of the composite outcome within one year (HR: 1.32; 95% CI 1.15–1.51; p < 0.001). The incorporation of low PMA into TRI-SCORE improved predictive performance for the composite outcome (area under the curve [AUC]: from 0.69 to 0.74; Supplemental Figure S3).

### Changes in NYHA class after TTVR

**Fig. 4:**
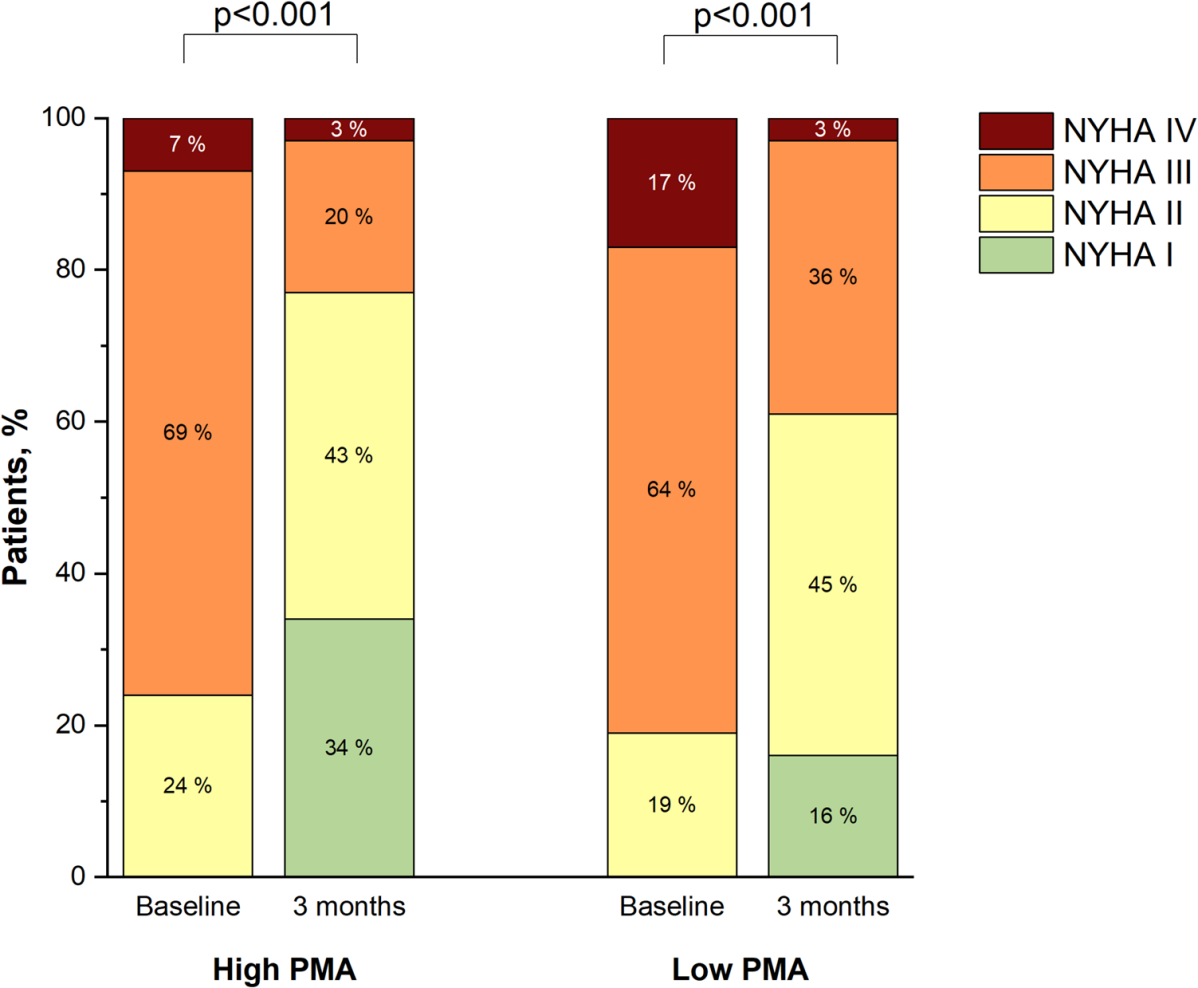
Changes in New York Heart Association class after transcatheter tricuspid valve repair

Data of the NYHA class data at the three-month follow-up was available in 101 patients (62%). Patients with low PMA had significant improvement in NYHA class from baseline to the three-month follow-up, as well as those with high PMA (Fig. 4). A reduction of the NYHA class ≥ one grade at the three-month follow-up was also comparable between the two groups (40% vs. 44%; p = 0.24). In contrast, patients with low PMA were more likely to have severe heart failure symptoms (i.e., NYHA III or IV) at the follow-up than those with high PMA (39% vs. 23%; p = 0.15).

## Discussion

In the present study, we investigated the association between PMA measured by CT and clinical outcomes in patients undergoing TTVR. The main findings can be summarized as follows:

1. Pre-procedural CT scans showed that 29% of patients undergoing TTVR had low PMA.
2. Procedural success and in-hospital mortality were comparable between patients with low and high PMA.
3. Low PMA was associated with a higher risk of all-cause mortality and heart failure hospitalization within one year after TTVR, independently of baseline characteristics.
4. Adding low PMA to TRI-SCORE improved its predictive performance for all-cause mortality and heart failure hospitalization within one year.
5. TTVR achieved an improvement of the NYHA class at the three-month follow-up, irrespective of PMA status.

Sarcopenia is a progressive skeletal muscle disorder that is associated with compromised functional capacity and impaired prognosis [22]. The assessment of muscle area using CT images may assist with the detection of sarcopenia [9, 12–14]. A reduction of PMA measured by CT images is a simple marker of sarcopenia with prognostic impacts in the setting of transcatheter interventions and cardiac surgeries [11, 13, 16]. In the present study, we measured PMA using pre-procedural CT images in patients undergoing TTVR. According to the previously shown cut-off value of PMA, approximately 30% of patients undergoing TTVR were considered as having low PMA. Patients with low PMA were more likely to be male and to have end-stage chronic kidney disease requiring hemodialysis than those with high PMA, resulting in a higher EuroSCORE II. These characteristics of patients with low PMA aligned with those in previous studies [23–25]. In contrast, most clinical demographics, including age and BMI, were comparable between patients with low and high PMA. This finding underscores the difficulty of identifying patients with low PMA from clinical information and supports the clinical relevance of CT assessment as a screening tool [26–28].

Patients with sarcopenia have been considered as high-risk population for cardiac surgery, including tricuspid valve surgery. The present study showed that TTVR achieved a high procedural success rate in patients with low PMA, without increasing the risk of in-hospital mortality. The risk of major or more bleeding, acute kidney injury, and pneumonia were also comparable between patients with low and high PMA. Furthermore, TTVR achieved post-procedural symptomatic improvements, independently of PMA. TTVR may offer a safe and effective treatment option to reduce TR in patients with sarcopenia.

Despite the procedural results, patients with low PMA had a significantly higher risk of all-cause mortality and heart failure hospitalization within one year after TTVR. This association was consistent after adjusting for baseline characteristics and TR reduction. This poor prognosis of patients with low PMA was mainly driven by all-cause mortality. Of note, patients with high PMA showed a remarkably low incidence of mortality within one year (less than 10%). Our findings indicate that the prognosis of patients with sarcopenia may be still poor even after TR reduction.

Given the negative impact of low PMA on prognosis after TTVR, the question can arise whether TTVR should be withheld from patients with sarcopenia. The decision to perform TTVR should be considered in patients with sarcopenia. However, the present study showed that the improvement in the NYHA class after TTVR similarly observed in patients with low PMA, as well as those with high PMA. The improvement of heart failure symptoms or quality of life is one of the major benefits of TTVR and may be associated with the improved clinical outcomes after TTVR [29]. Accordingly, given the safety and effectiveness in reducing TR, patients with sarcopenia could obtain benefits of TTVR and might be good candidates for the intervention. In addition, future analyses are needed to clarify the prognostic benefits of TTVR in patients with sarcopenia.

Accurate risk stratification is essential for appropriate patient selection for TTVR. TRI-SCORE is a dedicated risk score to predict in-hospital mortality after isolated tricuspid valve surgery [30]. Our prior work showed that TRI-SCORE was also effective in predicting clinical outcomes in patients undergoing TTVR [21]. Although it is widely applied to clinical practice, the TRI-SCORE does not include information about frailty and physical capacity of patients which have been considered as strong surgical risk factors. The present study showed that PMA assessed by CT images had an incremental value for predicting clinical outcomes after TTVR beyond TRI-SCORE. Assessment of PMA using pre-procedural CT images may help risk stratification and patient selection for TTVR.

According to current guidelines, diagnosing sarcopenia requires a comprehensive assessment that includes measurements of muscle strength and physical performance, such as grip strength and gait speed, in addition to low PMA [9]. It should be therefore acknowledged that assessments of muscle strength and physical performance were lacking in the present study. Nonetheless, the PMA measured by CT is strongly related to physical performance in the elderly population [31], and the CT assessment will be easily applied to the clinical practice, since the measurement of PMA is simple and has high reproducibility. Our work highlights that sarcopenia assessed by the PMA may be potentially associated with clinical outcomes in patients undergoing TTVR, and the CT assessment of PMA may refine the risk stratification in patients undergoing TTVR. To validate our findings, future studies incorporating the assessment of physical performance are needed in this population [32, 33].

### Limitations

Several limitations should be acknowledged in this study. Firstly, the retrospective and observational study design could introduce the potential for selection bias. The present study included only patients who underwent both pre-procedural CT and TTVR. Patients who were deemed not eligible for TTVR due to severe frailty or those who had any contraindications for CT might be excluded from the analysis. Although patients without pre-procedural CT images were likely to have severe heart failure symptoms, worse renal function, and impaired right ventricular function than those with pre-procedural CT images, most of profiles were comparable between the two groups (Supplemental Table S1). Therefore, the findings of our study would be applicable to clinical practice. Secondly, the cut-off values of PMA in our study were derived from a prior research by Mamane et al. [16], while established thresholds of PMA are still lacking. Finally, the changes in PMA after TTVR were not evaluated in the present study, which might affect the prognosis after TTVR. Further studies are necessary to investigate the impact of TTVR on sarcopenic status of the patients.

## Conclusion

Approximately 30% of patients undergoing TTVR were found to have low PMA. Procedural outcomes and post-procedural symptomatic improvements were comparable between patients with low and high PMA, while patients with low PMA had poor prognosis after TTVR. Although TTVR may be a safe therapeutic option to reduce TR and improve heart failure symptoms in patients with sarcopenia, the prognosis after TTVR remains poor in this population. Pre-procedural CT-based assessment of PMA may enhance risk stratification and support better clinical decision-making for TTVR.

## Supplementary Information

Below is the link to the electronic supplementary material.

Supplementary file1 (DOCX 292 KB)

## Data Availability

Most of the data generated or analysed during this study are included in the supplementary information files. All datasets used or analysed during the current study are available from the corresponding author on reasonable request.
