# Supplementary material for: Association between psoas muscle area and outcomes after transcatheter tricuspid valve repair
Source: Cardiovasc Interv Ther. 2025 May 27;40(3):679–88. doi: 10.1007/s12928-025-01136-3 (PMC12167307; doi:10.1007/s12928-025-01136-3)
Supplement: Supplementary file 1 — Supplementary file1 (DOCX 292 KB) [file 12928_2025_1136_MOESM1_ESM.docx]

**Supplemental materials**

**Supplemental Figure S1:** Study flowchart

**Supplemental Figure S2:** Incidences of clinical outcomes within one year

| 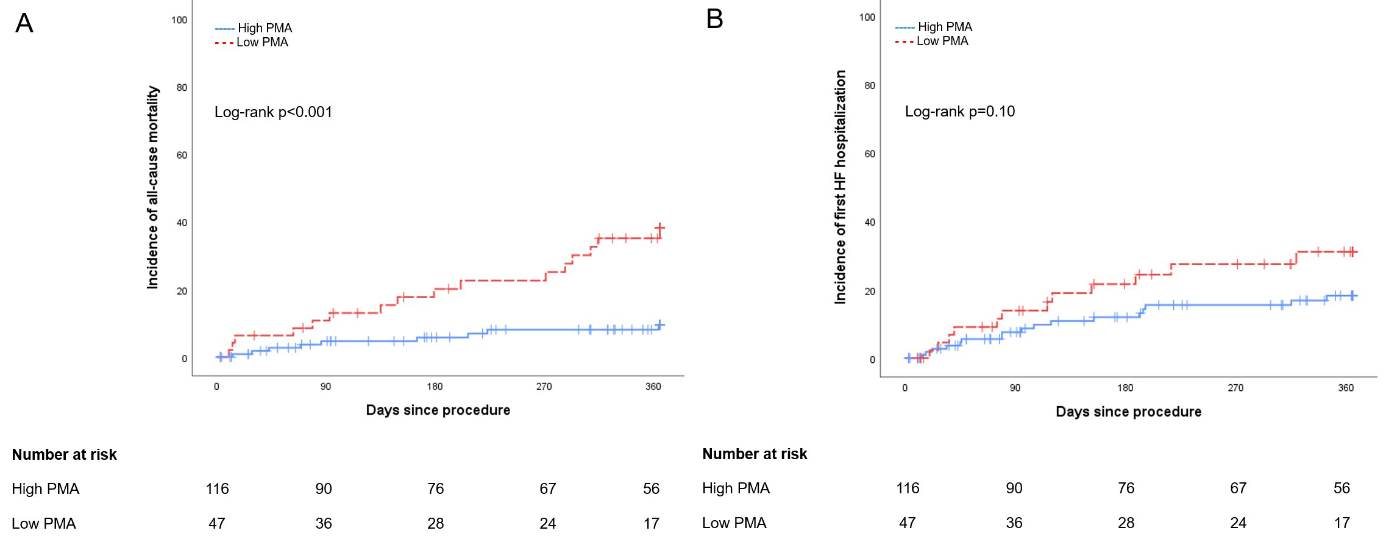 |
| --- |
| Incidences of all-cause mortality (A), hospitalization due to heart failure (B).  Legends: HF = Heart Failure. |

**Supplemental Figure S3:** Receiver-operating characteristic curve analysis comparing TRI-SCORE alone and the combination of low PMA with TRI-SCORE for predicting the composite outcome after TTVR.


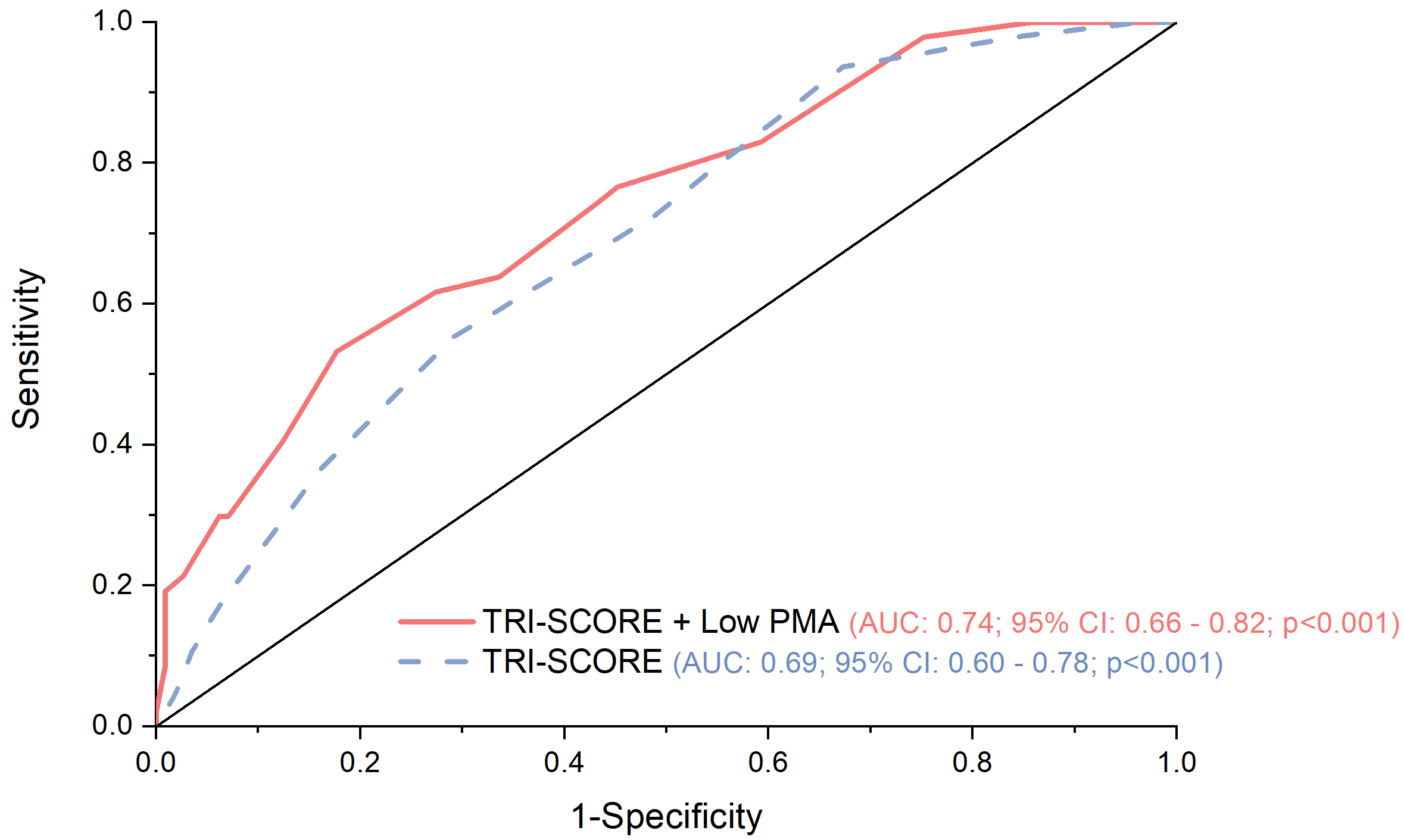


AUC = area under the curve.

**Supplemental Table S1:** Comparison of baseline characteristics between patients with and without pre-procedural CT images

|  | | Patients with CT | Patients without CT | p-value |
| --- | --- | --- | --- | --- |
|  |  | n= 163 | N=176 |  |
| Age, year | | 80 (76 – 84) | 79 (76 – 83) | 0.34 |
| Male | | 73 (45) | 80 (45) | 0.99 |
| BMI, kg/m^2^ | | 24.6 (22.0 – 27.2) | 25.7 (23.0 – 28.4) | 0.026 |
| EuroSCORE Ⅱ, % | | 5.5 (3.2 – 11.5) | 7.8 (4.8 – 13.3) | 0.002 |
| TRI-SCORE, point | | 5 (3 – 6) | 5 (4 – 6) | 0.21 |
| Diabetes mellitus | | 33 (20) | 46 (26) | 0.12 |
| Arterial hypertension | | 135 (83) | 150 (85) | 0.48 |
| Coronary artery disease | | 80 (49) | 94 (53) | 0.42 |
| Previous myocardial infarction | | 28 (17) | 40 (22) | 0.18 |
| Previous cardiac surgery | | 69 (42) | 93 (53) | 0.049 |
| Previous stroke | | 12 (7) | 22 (12) | 0.14 |
| Atrial fibrillation | | 150 (92) | 162 (92) | 0.99 |
| NYHA functional class | |  |  | 0.018 |
|  | II | 37 (23) | 23 (13) |  |
|  | III | 109 (67) | 123 (70) |  |
|  | IV | 16 (10) | 30 (17) |  |
| Cardiac implantable electrical device | | 38 (23) | 58 (33) | 0.042 |
| Hemoglobin, mg/dl | | 11.4 ± 1.9 | 11.6 ± 2.1 | 0.33 |
| eGFR, ml/min/m^2^ | | 51 (35 – 64) | 44 (33 – 60) | 0.046 |
| Hemodialysis | | 5 (3) | 4 (2) | 0.58 |
| NT-proBNP, pg/ml | | 2225 (1283 – 4094) | 1839 (1151 – 3751) | 0.26 |
| Furosemide equivalent dose, mg/day | | 40 (20 – 80) | 40 (20 – 80) | 0.98 |
| LVEF, % | | 57 (52 – 62) | 56 (50 – 62) | 0.28 |
| LV end-diastolic volume index, ml /m² | | 39 (28 – 51) | 39 (29 – 55) | 0.94 |
| LV end-systolic volume index, ml /m² | | 15 (11 – 24) | 17 (12 – 24) | 0.80 |
| RA area, cm^2^ | | 30 (24 – 35) | 28 (23 – 37) | 0.62 |
| Tricuspid annulus diameter, mm | | 44 ± 7 | 45 ± 8 | 0.038 |
| RV mid-ventricular diameter, mm | | 38 (31 – 43) | 37 (33 – 44) | 0.35 |
| Secondary TR | | 149 (91) | 140 (80) | 0.70 |
| Severity of TR | |  |  | 0.83 |
|  | Severe | 80 (49) | 67 (38) |  |
|  | Massive | 68 (42) | 82 (47) |  |
|  | Torrential | 15 (9) | 27 (15) |  |
| TAPSE, mm | | 19 ± 4.9 | 17.6 ± 5.0 | 0.046 |
| RVFAC, % | | 45 ± 10 | 41.7 ± 10.0 | 0.007 |
| SPAP, mmHg | | 46 ± 15 | 45 ± 15 | 0.92 |
| Values are either the number (%), mean ± SD, or median (interquartile range).  Abbreviations are shown in Table 1. | | | | |

**Supplemental Table S2:** Association of low psoas mass area (PMA) with the composite outcome within one year.

|  | Univariate analysis | | |
| --- | --- | --- | --- |
|  | HR | 95%CI | p-value |
| Low PMA | 2.52 | 1.42 - 4.47 | 0.002 |
| Age | 0.98 | 0.94 - 1.02 | 0.31 |
| Male sex | 1.70 | 0.95 - 3.02 | 0.07 |
| BMI, kg/m2 | 1.00 | 0.95 - 1.06 | 0.88 |
| eGFR, ml/min/m2 | 0.98 | 0.97 - 1.00 | 0.008 |
| LVEF, % | 0.97 | 0.95 - 0.99 | 0.02 |
| Smoking history | 1.57 | 0.85 - 2.91 | 0.15 |
| Diabetes mellitus | 1.23 | 0.64 - 2.37 | 0.54 |
| Hypertension | 1.22 | 0.55 - 2.72 | 0.63 |
| COPD | 2.08 | 1.10 - 3.95 | 0.03 |
| Hemodialysis | 1.15 | 0.28 - 4.75 | 0.85 |
| Coronary artery disease | 1.18 | 0.94 - 1.48 | 0.16 |
| Previous myocardial infarction | 1.77 | 0.93 - 3.35 | 0.08 |
| Previous cardiac surgery | 1.03 | 0.58 - 1.84 | 0.91 |
| Previous TIA/ stroke | 0.84 | 0.26 - 2.71 | 0.77 |
| Atrial fibrillation | 1.40 | 0.43 - 4.51 | 0.57 |
| NYHA functional class: III or IV | 1.08 | 0.54 - 2.18 | 0.83 |
| NT-proBNP, pg/ml | 1.00 | 1.00 - 1.00 | 0.13 |
| EuroSCOREII | 1.03 | 0.99 - 1.07 | 0.12 |
| TRI-SCORE | 1.32 | 1.15 - 1.51 | <0.001 |
| LV end-diastolic volume index, ml/m² | 1.01 | 0.99 - 1.03 | 0.27 |
| LV end-systolic volume index, ml/m² | 1.02 | 1.00 - 1.04 | 0.14 |
| Left atrial volume index, ml/m² | 1.00 | 0.98 - 1.02 | 0.92 |
| Right atrial area, cm² | 1.00 | 0.98 - 1.03 | 0.72 |
| Tricuspid annulus diameter, mm | 0.96 | 0.92 - 1.01 | 0.09 |
| RV basal diameter, mm | 1.01 | 0.98 - 1.04 | 0.53 |
| RV mid-ventricular diameter, mm | 1.04 | 1.01 - 1.07 | 0.02 |
| Secondary TR | 0.69 | 0.27 - 1.74 | 0.43 |
| Massive or torrential TR | 1.33 | 0.75 - 2.37 | 0.33 |
| TAPSE, mm | 0.92 | 0.86 - 0.98 | 0.007 |
| SPAP, mmHg | 1.00 | 0.98 - 1.02 | 0.88 |
| Abbreviations are shown in Table 1. | | | |

**Supplemental Table S3:** Association of low psoas mass area (PMA) with the composite outcome within one year.

|  | **Univariate analysis** | | | **Multivariable analysis** | | |
| --- | --- | --- | --- | --- | --- | --- |
|  | HR | 95%CI | p-value | HR | 95%CI | p-value |
| Low PMA | 2.52 | 1.42 – 4.47 | 0.002 | 2.35 | 1.32 – 4.17 | 0.004 |
| TRI-SCORE | 1.32 | 1.15 – 1.51 | <0.001 | 1.30 | 1.14 – 1.49 | <0.001 |
| Abbreviations are shown in Table 1. | | | | | | |
